# Anesthetic management of patients with sepsis/septic shock

**DOI:** 10.3389/fmed.2023.1150124

**Published:** 2023-03-23

**Authors:** Andrea Carsetti, Eva Vitali, Lucia Pesaresi, Riccardo Antolini, Erika Casarotta, Elisa Damiani, Erica Adrario, Abele Donati

**Affiliations:** ^1^Department of Biomedical Sciences and Public Health, Università Politecnica delle Marche, Ancona, Italy; ^2^Anesthesia and Intensive Care Unit, Azienda Ospedaliero Universitaria delle Marche, Ancona, Italy

**Keywords:** sepsis, septic shock, anesthesia, surgery, perioperative, infection, source control

## Abstract

Sepsis is defined as life-threatening organ dysfunction caused by a dysregulated host response to infection, while septic shock is a subset of sepsis with persistent hypotension requiring vasopressors to maintain a mean arterial pressure (MAP) of ≥65 mmHg and having a serum lactate level of >2 mmol/L, despite adequate volume resuscitation. Sepsis and septic shock are medical emergencies and time-dependent diseases with a high mortality rate for which early identification, early antibiotic therapy, and early source control are paramount for patient outcomes. The patient may require surgical intervention or an invasive procedure aiming to control the source of infection, and the anesthesiologist has a pivotal role in all phases of patient management. During the preoperative assessment, patients should be aware of all possible organ dysfunctions, and the severity of the disease combined with the patient's physiological reserve should be carefully assessed. All possible efforts should be made to optimize conditions before surgery, especially from a hemodynamic point of view. Anesthetic agents may worsen the hemodynamics of shock patients, and the anesthesiologist must know the properties of each anesthetic agent. All possible efforts should be made to maintain organ perfusion supporting hemodynamics with fluids, vasoactive agents, and inotropes if required.

## Introduction

Sepsis is defined as life-threatening organ dysfunction caused by a dysregulated host response to infection, while septic shock is a subset of sepsis with persisting hypotension requiring vasopressors to maintain a mean arterial pressure (MAP) of ≥65 mmHg and having a serum lactate level of >2 mmol/L, despite adequate volume resuscitation (1). The 30-day mortality rates for sepsis and septic shock have been recently estimated at 24.4 and 34.7%, respectively (2).

Concomitantly with resuscitation, a pillar of sepsis/septic shock management is source control (3). The patient must undergo diagnostic tests to identify the source of the infection and to determine if an invasive approach is needed to eliminate the source. Surgical intervention is needed, for example, in case of peritonitis or necrotizing fasciitis, surgical or percutaneous drainage is required for abscess, and nephrostomy or ureteral stenting is needed for hydronephrosis or urinary tract obstruction. In addition to source control, the invasive procedure also allows a sample for microbiological culture directly from the source of infection. Thus, the anesthesiologist may face a critically ill patient, the management of whom may be challenging. In this study, we review the peri-operative management of patients with sepsis/septic shock, describing the main issues of preoperative and intraoperative management.

## Preoperative management

Patients with sepsis/septic shock may need preoperative optimization. Frequently, the patients need an urgent surgical intervention to control the source of infection, and the preoperative assessment should be focused to identify and support organ dysfunction. Organ dysfunction may be assessed by the SOFA score, which considers Glasgow Coma Scale (GCS), PaO_2_/FIO_2_, and MAP, considering the need for vasoactive drugs, platelets count, bilirubin level, and creatinine/urine output (4).

Hemodynamic optimization must be performed before anesthesia induction and continued during the surgery. The balanced crystalloid solution should be used to correct hypovolemia. Current guidelines suggest administering up to 30 ml/kg in the first 3 h (3) even if a personalized approach based on the fluid challenge technique is considered appropriate (5). An increase in the stroke volume (SV) to >10–15% after the infusion of 3 ml/kg of crystalloid over 5 min defines a responsive patient, and the test may be repeated until the patient is responsive (6). However, SV monitoring may not be always available, especially in the early phase of assessment and treatment. An increase in systolic and mean arterial pressures have been assessed as alternative parameters to consider for fluid responsiveness evaluation, but they have not shown good sensitivity and specificity (7). An increase in pulse pressure of >10% has been demonstrated as a more reliable parameter to consider (7).

If the patient is still hypotensive after initial fluid therapy, a vasoactive drug should be early started while volemia is still under optimization (8). Noradrenaline is the vasoconstrictor of choice (3) and may be initially infused also in the peripheral veins (3, 9). No major complication has been reported when an antecubital vein has been used, avoiding the smaller distal ones (10–14). This may be a reasonable approach in the meantime central venous access is established.

Cardiac function should be assessed to identify arrhythmias or septic cardiomyopathy. Septic cardiomyopathy, defined as any cardiac dysfunction (left ventricular systolic or diastolic dysfunction or right ventricular dysfunction) unrelated to ischemia (15, 16), may be present in a variable percentage of patients, ranging from 10 to 70% (15), and may significantly contribute to hemodynamic instability and hypoperfusion. Echocardiography has a pivotal role to assess cardiac function. Biomarkers such as troponin or BNP rise in relation to cardiac injury even if different mechanisms of release may be identified during sepsis (e.g., inflammation, myocardial wall stress, and renal impairment) reflecting the severity of critical illness and other organ dysfunctions independent of the cardiac cells' death (15).

Optimizing oxygen delivery, red blood cell (RBC) transfusion may be needed to maintain hemoglobin (Hb) levels between 7 and 9 g/dl (3, 17).

According to the source of the infection and the presence of lung inflammation, the patient may be dyspneic and hypoxic. An increased work of breathing and tachypnea may be due to the compensatory mechanism of metabolic acidosis, while hypoxia may be present if acute respiratory distress syndrome (ARDS) coexists. Physical examination, SpO_2_, EGA, and chest imaging (chest X-ray and CT scan) are fundamental to identifying lung injury.

A careful assessment of renal function must be performed as sepsis/septic shock may be responsible for acute kidney injury. Urea and creatinine levels must be assessed, as well as diuresis and electrolytes.

Finally, coagulopathy may be an important issue in patients with sepsis/septic shock. It may manifest as a mild/moderate alteration of the coagulation test (INR and PTT) and/or thrombocytopenia up to the most severe scenario of disseminated intravascular coagulopathy (DIC). The International Society on Thrombosis and Hemostasis has established the criteria for overt DIC and sepsis-induced coagulopathy (SIC) (Table 1) (18). Rotational thromboelastography (TEG) and thromboelastometry (ROTEM) may have a role in identifying alterations in coagulation in sepsis (19, 20) and an early prediction of DIC (21). An hypocoagulability state may be detected by TEG/ROTEM also in patients with normal INR/PTT values and is associated with increased mortality (22). In patients with DIC and active bleeding or at high risk of bleeding (e.g., patients who undergo an invasive procedure), fresh-frozen plasma (FFP) (10–15 mL/kg) should be administered (23, 24). To avoid fluid overload, factor concentrates such as prothrombin complex concentrate might be considered as an alternative (23, 24). Fibrinogen concentrate or cryoprecipitate may be used to treat severe hypo-fibrinogenemia (< 1 g/L) (23, 24). The transfusion of platelets should be considered for patients with active bleeding or at high risk of bleeding who have a platelet count of < 50 × 10^9^/L (23, 24).

**Table 1:** ISTH overt DIC and SIC scoring systems.

	**Score**	**Overt DIC**	**SIC**
Platelet count (× 10^9^/L)	2	< 50	< 100
	1	50–100	100–150
FDP/D-dimer	3	Strong increase	
	2	Moderate increase	
PT (PT ratio)	2	≥6	(>1.4)
	1	3–5	(1.2–1.4)
Fibrinogen (g/ml)	1	< 100	-
SOFA score	2	-	≥2
	1	-	1
Total score		≥5	≥4

DIC, disseminated intravascular coagulation; FDP, fibrin/fibrinogen degradation products; PT, prothrombin time; SIC, sepsis-induced coagulopathy; SOFA, sequential organ failure assessment.

Concurrently with resuscitation, broad-spectrum antibiotics should be started within 1 h from the suspect of sepsis/septic shock after culture sampling (3). The antibiotic choice should be based on the site of suspected infection, the risk factors for multidrug-resistant microorganisms, the severity of the disease, and the local epidemiology.

## Intraoperative management

Considering the emergency/urgency indication for surgical procedures in patients with sepsis/septic shock, the rapid sequence induction (RSI) technique should be used for the induction of general anesthesia.

Patients with shock have a greater hemodynamic and nervous system sensitivity to anesthetic agents, and a lower anesthetic dose is required to exert the desired effect (25). Patients with sepsis may have unstable hemodynamics, and the induction of anesthesia may be responsible for severe hypotension due to vasodilation and myocardial depression. All possible efforts should be made to optimize hemodynamics before induction. In addition to the standard monitoring, patients with sepsis/septic shock must have invasive arterial monitoring and a central venous catheter. Preload should be optimized, and the vasopressor is readily available or ongoing to prevent the inevitable adverse effects of induction agents (e.g., vasodilation). Patients with hypovolemia may present severe hypotension with any anesthetic drugs due to the inhibition of compensatory sympathetic stimulation. Zausig et al. (26) tested the dose–response direct cardiac effects of induction agents in an isolated septic rat heart model, and they showed that the cardiac work dysfunction was highest for propofol (−50%), followed by midazolam (−38%), etomidate (−17%), and s(+)-ketamine (−6%). The titration of the dose is paramount to identify the lower effective dose and limits the adverse hemodynamic effects.

Ketamine and etomidate are the agents of choice for their limited hemodynamic effects compared to other anesthetic drugs. The myocardial depression induced by ketamine is counterbalanced by its stimulation in catecholamine release, which however may be blunted in critically ill patients (27). Experimental evidence suggests that ketamine may have an anti-inflammatory effect reducing the production and release of cytokines in endotoxemia *in vitro* (28–31). There is scarce evidence for which is the superior hypnotic agent between ketamine and etomidate. A retrospective study showed that etomidate was associated with less hypotension than ketamine during intubation of septic patients in the emergency department (32). Even if etomidate has adrenal suppression properties that may be responsible for an increased incidence of hypotension within the first 24 h after induction (33), a single bolus for induction is not associated with increased morbidity and mortality in critically ill patients, including those with sepsis (34). A reduced dose and the administration of adjuvants may be considered to reduce the adverse effects of induction agents. Adding lidocaine to a reduced dose of ketamine, for example, is associated with fewer episodes of hypotension in patients with septic shock (35). Short-acting opioids (e.g., fentanyl, alfentanil, and remifentanil) also allow a reduction of the hypnotic agent.

Propofol may have a greater impact on patients with unstable hemodynamics due to vasodilation and myocardial depression. When used, it must be slowly titrated to find the lowest effective dose. Thus, its role in the RSI of shocked patients is limited (25). Even if preclinical trials have shown a potential anti-inflammatory effect of propofol, it increased TNF-α responses caused by LPS-stimulated human blood *in vitro* (36) and stimulated the production of TNF-α, interleukin-1, and interleukin-6 in critically ill surgical patients (37). Thus, the anti-inflammatory effects of propofol in endotoxemia remain unclear.

Midazolam may be an alternative induction agent. Even if it has fewer hemodynamic effects than propofol, the high protein binding and slow kinetic limit its use for RSI (25).

There is no evidence for the technique of choice for the maintenance of general anesthesia (volatile vs. intravenous anesthesia). Volatile anesthetics have immunomodulatory effects including inhibitory effects on neutrophil function, decreased lymphocyte proliferation, and suppressed cytokine release from peripheral blood mononuclear cells (38–40). However, the evidence of the effects of anesthetics on the immune system is mainly derived from *in vitro*/experimental studies. The anesthesiologist should know that sepsis/septic shock reduces the MAC for volatile anesthetics (41–43). Moreover, severe lung dysfunction may make it difficult to maintain a stable brain concentration of inhaled drugs (44). Monitoring the depth of anesthesia using the processed EEG may be useful to avoid drug overdose as well as the risk of awareness.

For appropriate intraoperative hemodynamic management, advanced hemodynamic monitoring may give important information and guidance for therapy and should be implemented if not already applied for the management of the preoperative phase. A mini-invasive hemodynamic system based on pulse contour analysis for cardiac output (CO) estimation should be used, and the choice of calibrated vs. uncalibrated systems depends on the severity of the shock. The reliability of uncalibrated systems is limited in patients with a high dose of vasoactive drugs or in case of rapid fluctuation of vasomotor tone. Central venous pressure (CVP) has a limited role to guide fluid administration (45). Dynamic indexes of fluid responsiveness (pulse pressure variation [PPV] and stroke volume variation [SVV]) should be considered to anticipate the patient's response to a fluid bolus. However, the limits for their applicability must be known, including arrhythmia, tidal volume lower than 8 ml/kg, low pulmonary compliance, intra-abdominal hypertension, and right ventricular dysfunction (46). Often, a low tidal volume of 6 ml/kg is applied as a protective lung ventilation strategy, and a tidal volume test may be performed temporarily increasing Vt to 8 ml/kg for a few breaths aiming to assess a dynamic test of fluid responsiveness. An increase in PPV or SVV of 3.5 and 2.5%, respectively, after the increase in tidal volume from 6 to 8 ml/kg are indicative of fluid responsiveness (47). Alternatively, an end respiratory occlusion test may be performed for 15–20 s, indicating fluid responsiveness if CO increases by at least 5% (48). When a large amount of fluid is required for preload optimization, albumin should be considered (3). Other types of colloids (e.g., gelatines and hydroxyethyl starch) are currently contraindicated (3). In case of refractory hypotension despite fluid resuscitation and a high dose of noradrenaline, vasopressin may be added (3). A low dose of hydrocortisone (200 mg/day iv) should also be considered in patients with septic shock and an ongoing requirement for vasopressor (3). A low CO associated with hypoperfusion despite adequate fluid resuscitation and afterload optimization may indicate the need for an inotropic agent. Dobutamine is the drug of choice (3). A central venous catheter is mandatory when vasoactive drugs are needed, and central venous oxygen saturation (ScvO_2_) is also a useful parameter to assess the adequacy of tissue perfusion, with arterial lactate and Pv-aCO_2_. Lactate > 2 mmol/l, ScvO_2_ < 70%, and Pv-aCO_2_ > 6 mmHg are signs of tissue hypoperfusion (49). Patients' position and surgical technique may also have an impact on hemodynamics. Preload and CO may be severely reduced in patients with hypovolemia undergoing laparoscopic surgery with pneumoperitoneum (for the vena cava compression) and/or anti-Trendelenburg position (for blood pooled in the capacitance veins of the pelvis and the legs) (50, 51). During surgery, bleeding may be responsible for further hemodynamic derangement. Transfusion may be required to maintain the Hb target.

Protective ventilation strategies should be applied during mechanical ventilation. A low tidal volume (6–8 ml/kg predicted body weight) is recommended (52), and the lowest possible FIO_2_ to achieve SpO_2_ ≥ 94% should be used (52). Patients with hypoxemias may benefit from recruitment maneuvers and an appropriate positive end-expiratory pressure (PEEP) setting. However, their hemodynamic effects are significant in patients with hypovolemia, and severe hypotension may occur. If needed, preload should be previously assessed and optimized. Obese patients and pneumoperitoneum decrease abdominal/chest compliance and a higher value of PEEP may be needed to avoid atelectasis. For the same reason, a higher plateau pressure (Pplat) may be tolerable. The PEEP level should be titrated to obtain the lowest value of driving pressure (Pplat-PEEP) for the desired tidal volume (52, 53).

Depending on the duration of the procedure, repeated doses of antibiotics may be needed during surgery. Altered pharmacokinetics and pharmacodynamics must be considered, as patients with sepsis usually have a greater volume of distribution for hydrophilic drugs and altered organ function (54). Thus, a higher dose of hydrophilic drugs may be required.

## Postoperative considerations

Intensive care unit admission is often required after source control for patients with sepsis/septic shock. The patient may still be hypoperfused and hemodynamically unstable, requiring organ support. Moreover, further interventions may be required subsequently, e.g., in case of abdominal infection managed with an open abdomen requiring revision or daily necrosectomy to face necrotizing fasciitis. Organ supports like mechanical ventilation and renal replacement therapy may be required postoperatively.

## Conclusion

Sepsis and septic shock are medical emergencies and time-dependent diseases for which early identification, early antibiotic therapy, and early source control are paramount for patient outcomes. The patient may require surgical intervention or an invasive procedure aiming to control the source of infection, and the anesthesiologist has a pivotal role in all phases of patient management. During the preoperative assessment, the patients should be aware of all possible organ dysfunctions, and the severity of the disease combined with the patient's physiological reserve should be carefully assessed. The time for preoperative assessment and optimization is usually limited as the procedure is often urgent/emergent. Nevertheless, all possible efforts should be made to optimize conditions before surgery, especially from a hemodynamic point of view. Anesthetic agents may worsen the hemodynamics of patients with shock, and the anesthesiologist must know the properties of each anesthetic agent. All possible efforts should be made to maintain organ perfusion supporting hemodynamics with fluids, vasoactive agents, and inotropes if required.

## Author contributions

AC conceived and wrote the manuscript. EV, LP, RA, EC, ED, EA, and AD substantially contributed to the manuscript revision. All authors contributed to the article and approved the submitted version.

## References

[1] Singer M Deutschman CS Seymour CW Shankar-Hari M Annane D Bauer M. The third international consensus definitions for sepsis and septic shock (Sepsis-3). JAMA. (2016) 315:801. 10.1001/jama.2016.0287 26903338 PMC4968574

[2] Bauer M Gerlach H Vogelmann T Preissing F Stiefel J Adam D. Mortality in sepsis and septic shock in Europe, North America and Australia between 2009 and 2019- results from a systematic review and meta-analysis. Crit Care. (2020) 24:1–9. 10.1186/s13054-020-02950-2 32430052 PMC7236499

[3] Evans L Rhodes A Alhazzani W Antonelli M Coopersmith CM French C. Surviving sepsis campaign: international guidelines for management of sepsis and septic shock 2021. Intensive Care Med. (2021) 47:1181–247. 10.1007/s00134-021-06506-y 34599691 PMC8486643

[4] Vincent JL De Mendonça A Cantraine F Moreno R Takala J Suter PM. Use of the SOFA score to assess the incidence of organ dysfunction/failure in intensive care units: results of a multicenter, prospective study. Working group on “sepsis-related problems” of the European Society of Intensive Care Medicine. Crit Care Med. (1998) 26:1793–800. 10.1097/00003246-199811000-00016 9824069

[5] Malbrain MLNG Van Regenmortel N Saugel B De Tavernier B Van Gaal PJ Joannes-Boyau O. Principles of fluid management and stewardship in septic shock: it is time to consider the four D's and the four phases of fluid therapy. Ann Intensive Care. (2018) 8:1–16. 10.1186/s13613-018-0402-x 29789983 PMC5964054

[6] Cecconi M Parsons AK Rhodes A. What is a fluid challenge? Curr Opin Crit Care. (2011) 17:290–5. 10.1097/MCC.0b013e32834699cd 21508838

[7] Ait-Hamou Z Teboul JL Anguel N Monnet X. How to detect a positive response to a fluid bolus when cardiac output is not measured? Ann Intensive Care. (2019) 9:1–9. 10.1186/s13613-019-0612-x 31845003 PMC6915177

[8] Ospina-Tascón GA Hernandez G Alvarez I Calderón-Tapia LE Manzano-Nunez R Sánchez-Ortiz AI. Effects of very early start of norepinephrine in patients with septic shock: a propensity score-based analysis. Crit Care. (2020) 24:1–11. 10.1186/s13054-020-2756-3 32059682 PMC7023737

[9] Carsetti A Bignami E Cortegiani A Donadello K Donati A Foti G. Good clinical practice for the use of vasopressor and inotropic drugs in critically ill patients: state-of-the-art and expert consensus. Minerva Anestesiol. (2021) 87:714–32. 10.23736/S0375-9393.20.14866-1 33432794

[10] Delaney A Finnis M Bellomo R Udy A Jones D Keijzers G. Initiation of vasopressor infusions via peripheral versus central access in patients with early septic shock: A retrospective cohort study. Emerg Med Australas. (2020) 32:210–9. 10.1111/1742-6723.13394 31599084

[11] Medlej K Kazzi AA El Hajj Chehade A Saad Eldine M Chami A Bachir R. Complications from administration of vasopressors through peripheral venous catheters: an observational study. J Emerg Med. (2018) 54:47–53. 10.1016/j.jemermed.2017.09.007 29110979

[12] Cardenas-Garcia J Schaub KF Belchikov YG Narasimhan M Koenig SJ Mayo PH. Safety of peripheral intravenous administration of vasoactive medication. J Hosp Med. (2015) 10:581–5. 10.1002/jhm.2394 26014852

[13] Loubani OM Green RS. A systematic review of extravasation and local tissue injury from administration of vasopressors through peripheral intravenous catheters and central venous catheters. J Crit Care. (2015) 30:653.e9–653.e17. 10.1016/j.jcrc.2015.01.014 25669592

[14] Simons C. The safety and efficacy of peripherally administered norepinephrine during the perioperative period. AANA J. (2022) 90:387–95. 36173798

[15] Carbone F Liberale L Preda A Schindler TH Montecucco F. Septic cardiomyopathy: from pathophysiology to the clinical setting. Cells. (2022) 11:2833. 10.3390/cells11182833 36139408 PMC9496713

[16] Beesley SJ Weber G Sarge T Nikravan S Grissom CK Lanspa MJ. Septic cardiomyopathy. Crit Care Med. (2018) 46:625–34. 10.1097/CCM.0000000000002851 29227368

[17] Vlaar AP Oczkowski S de Bruin S Wijnberge M Antonelli M Aubron C. Transfusion strategies in non-bleeding critically ill adults: a clinical practice guideline from the European Society of Intensive Care Medicine. Intensive Care Med. (2020) 46:673–96. 10.1007/s00134-019-05884-8 31912207 PMC7223433

[18] Iba T Levy JH Warkentin TE Thachil J van der Poll T Levi M. Diagnosis and management of sepsis-induced coagulopathy and disseminated intravascular coagulation. J Thromb Haemost. (2019) 17:1989–94. 10.1111/jth.14578 31410983

[19] Müller MC Meijers JCM Vroom MB Juffermans NP. Utility of thromboelastography and/or thromboelastometry in adults with sepsis: a systematic review. Crit Care. (2014) 18:1–11. 10.1186/cc13721 24512650 PMC4056353

[20] Luo C Hu H Gong J Zhou Y Chen Z Cai S. The value of thromboelastography in the diagnosis of sepsis-induced coagulopathy. Clin Appl Thromb Hemost. (2020) 26:1076029620951847. 10.1177/1076029620951847 32870718 PMC7469719

[21] Kim SM Kim S Il Yu G Kim JS Hong SI Chae B. Role of thromboelastography as an early predictor of disseminated intravascular coagulation in patients with septic shock. J Clin Med. (2020) 9:1–11. 10.3390/jcm9123883 33260354 PMC7760761

[22] Kim SM Kim S Il Yu G Kim JS Hong SI Chae B. Role of thromboelastography in the evaluation of septic shock patients with normal prothrombin time and activated partial thromboplastin time. Sci Rep. (2021) 11:11833. 10.1038/s41598-021-91221-3 34088928 PMC8178375

[23] Levi M Toh CH Thachil J Watson HG. Guidelines for the diagnosis and management of disseminated intravascular coagulation. Br J Haematol. (2009) 145:24–33. 10.1111/j.1365-2141.2009.07600.x 19222477

[24] Di Nisio M Baudo F Cosmi B D'Angelo A De Gasperi A Malato A. Diagnosis and treatment of disseminated intravascular coagulation: guidelines of the Italian Society for Haemostasis and Thrombosis (SISET). Thromb Res. (2012) 129:1–8. 10.1016/j.thromres.2011.08.028 21930293

[25] Morris C Perris A Klein J Mahoney P. Anaesthesia in haemodynamically compromised emergency patients: does ketamine represent the best choice of induction agent? Anaesthesia. (2009) 64:532–9. 10.1111/j.1365-2044.2008.05835.x 19413824

[26] Zausig YA Busse H Lunz D Sinner B Zink W Graf BM. Cardiac effects of induction agents in the septic rat heart. Crit Care. (2009) 13:1–8. 10.1186/cc8038 19737388 PMC2784361

[27] Waxman K Shoemaker W Lippmann M. Cardiovascular effects of anesthetic induction with ketamine. Anesth Analg. (1980) 59:355–8. 10.1213/00000539-198005000-00007 7189381

[28] Kawasaki T Ogata M Kawasaki C Ogata JI Inoue Y Shigematsu A. Ketamine suppresses proinflammatory cytokine production in human whole blood in vitro. Anesth Analg. (1999) 89:665–9. 10.1213/00000539-199909000-00024 10475301

[29] Yu M Shao D Liu J Zhu J Zhang Z Xu J. Effects of ketamine on levels of cytokines, NF-kappaB and TLRs in rat intestine during CLP-induced sepsis. Int Immunopharmacol. (2007) 7:1076–82. 10.1016/j.intimp.2007.04.003 17570324

[30] Kawasaki C Kawasaki T Ogata M Nandate K Shigematsu A. Ketamine isomers suppress superantigen-induced proinflammatory cytokine production in human whole blood. Can J Anaesth. (2001) 48:819–23. 10.1007/BF03016701 11546726

[31] Sun J Wang XD Liu H Xu JG. Ketamine suppresses intestinal NF-kappa B activation and proinflammatory cytokine in endotoxic rats. World J Gastroenterol. (2004) 10:1028–31. 10.3748/wjg.v10.i7.1028 15052687 PMC4717093

[32] Mohr NM Pape SG Runde D Kaji AH Walls RM Brown CA. Etomidate use is associated with less hypotension than ketamine for emergency department sepsis intubations: A NEAR cohort study. Acad Emerg Med. (2020) 27:1140–9. 10.1111/acem.14070 32602974 PMC8711033

[33] Van Berkel MA Exline MC Cape KM Ryder LP Phillips G Ali NA. Increased incidence of clinical hypotension with etomidate compared to ketamine for intubation in septic patients: A propensity matched analysis. J Crit Care. (2017) 38:209–14. 10.1016/j.jcrc.2016.11.009 27974285

[34] Jabre P Combes X Lapostolle F Dhaouadi M Ricard-Hibon A Vivien B. Etomidate versus ketamine for rapid sequence intubation in acutely ill patients: a multicentre randomised controlled trial. Lancet (London, England). (2009) 374:293–300. 10.1016/S0140-6736(09)60949-1 19573904

[35] Fathy S Hasanin A Mostafa M Ramzy E Sarhan K Almenesey T. The benefit of adding lidocaine to ketamine during rapid sequence endotracheal intubation in patients with septic shock: A randomised controlled trial. Anaesthesia, Crit care pain Med. (2021) 40:100731. 10.1016/j.accpm.2020.06.017 32898698

[36] Larsen B Hoff G Wilhelm W Buchinger H Wanner GA Bauer M. Effect of intravenous anesthetics on spontaneous and endotoxin-stimulated cytokine response in cultured human whole blood. Anesthesiology. (1998) 89:1218–27. 10.1097/00000542-199811000-00023 9822011

[37] Helmy SAK Al-Attiyah RJ. The immunomodulatory effects of prolonged intravenous infusion of propofol versus midazolam in critically ill surgical patients. Anaesthesia. (2001) 56:4–8. 10.1046/j.1365-2044.2001.01713.x 11167428

[38] Cruz FF Rocco PRM Pelosi P. Anti-inflammatory properties of anesthetic agents. Crit Care. (2017) 21:1–7. 10.1186/s13054-017-1645-x 28320449 PMC5359894

[39] Lee YM Song BC Yeum KJ. Impact of Volatile Anesthetics on Oxidative Stress and Inflammation. Biomed Res Int. (2015) 2015:242709. 10.1155/2015/242709 26101769 PMC4458520

[40] Kurosawa S Kato M. Anesthetics, immune cells, and immune responses. J Anesth. (2008) 22:263–77. 10.1007/s00540-008-0626-2 18685933

[41] Gill R Martin C McKinnon T Lam C Cunningham D Sibbald WJ. Sepsis reduces isoflurane MAC in a normotensive animal model of sepsis. Can J Anaesth. (1995) 42:631–5. 10.1007/BF03011885 7554004

[42] Allaouchiche B Duflo F Tournadre JP Debon R Chassard D. Influence of sepsis on sevoflurane minimum alveolar concentration in a porcine model. Br J Anaesth. (2001) 86:832–6. 10.1093/bja/86.6.832 11573592

[43] Allaouchiche B Dufo F Debon R Tournadre JP Chassard D. Influence of sepsis on minimum alveolar concentration of desflurane in a porcine model. Br J Anaesth. (2001) 87:280–3. 10.1093/bja/87.2.280 11493502

[44] Eissa D Carton EG Buggy DJ. Anaesthetic management of patients with severe sepsis. Br J Anaesth. (2010) 105:734–43. 10.1093/bja/aeq305 21030391

[45] Osman D Ridel C Ray P Monnet X Anguel N Richard C. Cardiac filling pressures are not appropriate to predict hemodynamic response to volume challenge. Crit Care Med. (2007) 35:64–8. 10.1097/01.CCM.0000249851.94101.4F 17080001

[46] Carsetti A Cecconi M Rhodes A. Fluid bolus therapy: monitoring and predicting fluid responsiveness. Curr Opin Crit Care. (2015) 21:388–94. 10.1097/MCC.0000000000000240 26348418

[47] Myatra SN Prabu NR DIvatia JV Monnet X Kulkarni AP Teboul JL. The changes in pulse pressure variation or stroke volume variation after a “tidal volume challenge” reliably predict fluid responsiveness during low tidal volume ventilation. Crit Care Med. (2017) 45:415–21. 10.1097/CCM.0000000000002183 27922879

[48] Monnet X Osman D Ridel C Lamia B Richard C Teboul JL. Predicting volume responsiveness by using the end-expiratory occlusion in mechanically ventilated intensive care unit patients. Crit Care Med. (2009) 37:951–6. 10.1097/CCM.0b013e3181968fe1 19237902

[49] Cecconi M De Backer D Antonelli M Beale R Bakker J Hofer C. Consensus on circulatory shock and hemodynamic monitoring. Care Med. (2014) 40:1795–815. 10.1007/s00134-014-3525-z 25392034 PMC4239778

[50] Rist M Hemmerling TM Rauh R Siebzehnrübl E Jacobi KE. Influence of pneumoperitoneum and patient positioning on preload and splanchnic blood volume in laparoscopic surgery of the lower abdomen. J Clin Anesth. (2001) 13:244–9. 10.1016/S0952-8180(01)00242-2 11435046

[51] Hayden P Cowman S. Anaesthesia for laparoscopic surgery. Contin Educ Anaesth Crit Care Pain. (2011) 11:177–80. 10.1093/bjaceaccp/mkr027

[52] Young CC Harris EM Vacchiano C Bodnar S Bukowy B Elliott RRD. Lung-protective ventilation for the surgical patient: international expert panel-based consensus recommendations. Br J Anaesth. (2019) 123:898–913. 10.1016/j.bja.2019.08.017 31587835

[53] Neto AS Hemmes SNT Barbas CSV Beiderlinden M Fernandez-Bustamante A Futier E. Association between driving pressure and development of postoperative pulmonary complications in patients undergoing mechanical ventilation for general anaesthesia: a meta-analysis of individual patient data. Lancet Respir Med. (2016) 4:272–80. 10.1016/S2213-2600(16)00057-6 26947624

[54] Tsai D Lipman J Roberts JA. Pharmacokinetic/pharmacodynamic considerations for the optimization of antimicrobial delivery in the critically ill. Curr Opin Crit Care. (2015) 21:412–20. 10.1097/MCC.0000000000000229 26348420

